# MicroRNA-361-Mediated Inhibition of HSP90 Expression and EMT in Cervical Cancer Is Counteracted by Oncogenic lncRNA NEAT1

**DOI:** 10.3390/cells9030632

**Published:** 2020-03-05

**Authors:** Daozhi Xu, Peixin Dong, Ying Xiong, Junming Yue, Yosuke Konno, Kei Ihira, Noriko Kobayashi, Yukiharu Todo, Hidemichi Watari

**Affiliations:** 1 Department of Obstetrics and Gynecology, Hokkaido University School of Medicine, Hokkaido University, Sapporo 060-0817, Japan; xudaozhi87@yahoo.co.jp (D.X.); konsuke013@gmail.com (Y.K.); ihey0610@huhp.hokudai.ac.jp (K.I.); norikingyo@med.hokudai.ac.jp (N.K.); 2 Department of Gynecology, State Key Laboratory of Oncology in South China, Sun Yat-sen University Cancer Center, Guangzhou 510275, China; tdken999@163.com; 3 Department of Pathology and Laboratory Medicine, University of Tennessee Health Science Center, Memphis, TN 38163, USA; jyue@uthsc.edu; 4 Center for Cancer Research, University of Tennessee Health Science Center, Memphis, TN 38163, USA; 5 Division of Gynecologic Oncology, National Hospital Organization, Hokkaido Cancer Center, Sapporo 060-0042, Japan; yukiharu@sap-cc.go.jp

**Keywords:** microRNA-361, EMT, HSP90AA1, long non-coding RNA, NEAT1, cervical cancer

## Abstract

Epithelial-mesenchymal transition (EMT) is a key process contributing to cervical cancer (CC) metastasis, and microRNAs (miRNAs) modulate the expression of genes implicated in EMT. However, the accurate role of miR-361 in CC-associated EMT and the mechanisms underlying its function in CC remains largely unknown. The functional roles of miR-361 in CC cells were explored by a series of cell functional assays. Luciferase reporter assays were used to demonstrate the potential interaction between miR-361, HSP90, and long non-coding RNA (lncRNA) NEAT1. We detected a reduction of miR-361 expression in CC tissues compared with normal tissues, and miR-361 overexpression inhibited invasion and EMT phenotypes of CC cells by directly targeting a key EMT activator HSP90. Additionally, we detected significantly higher levels of HSP90 in CC tissues compared with normal tissues, and high expression of HSP90 predicted a poorer prognosis. We further identified NEAT1 as a significantly upregulated lncRNA in CC tissues and high expression of NEAT1 was associated with worse survival in CC patients. NEAT1 directly repressed miR-361 expression and played an oncogenic role in CC cell invasion and sphere formation. Conclusions: These results demonstrated that miR-361 directly targets HSP90 to inhibit the invasion and EMT features, and NEAT1 functions as an oncogenic lncRNA that suppresses miR-361 expression and induces EMT and sphere formation in CC cells, thus providing critical insights into the molecular pathways operating in this malignancy.

## 1. Introduction 

Cervical cancer is one of the most common malignancies in women worldwide, with a 5-year relative survival rate of 16.5% for metastatic cervical cancer [1]. Understanding the biology of metastatic cervical cancer cells will allow the development of novel therapeutic approaches aimed at improving the survival rates of patients with cervical cancer. 

Cancer metastasis begins with the process of epithelial-mesenchymal transition (EMT), which converts well-polarized epithelial cells to non-polarized mesenchymal cells that acquire motility and invasion properties and exhibit cancer stem cell-like properties [2]. Therefore, EMT has been shown to play a role in the tumorigenic and metastatic process. EMT is triggered by EMT-activating transcription factors, such as SNAIL, TWIST, and ZEB families [2]. Also, several oncogenes (including *MCL-1*, *Oct-4*, and *SOX-2*) have been shown to contribute to the metastatic potential of tumor cells through the promotion of EMT [3,4,5,6]. However, the molecular mechanisms underlying the regulation of EMT in cervical cancer cells are still largely unknown. 

The 90-kDa heat shock proteins (HSP90s) are abundant molecular chaperones, accounting for 1–2% of all proteins in the cell [7,8]. There are two major HSP90 isoforms, namely HSP90α (the stress-inducible isoform encoded by *HSP90AA1*) and HSP90β (the constitutively expressed isoform encoded by *HSP90AB1*) [7,8]. In an ATP-bound state, HSP90α (referred to as HSP90 in this study) adopts a closed conformation and forms a mature complex with co-chaperones to indirectly promote tumor progression by protecting and stabilizing its client proteins, such as mutated p53, HER-2, EGFR, BRAF, AKT, MET, VEGFR, FLT3, androgen/estrogen receptors, HIF-1αn and hTERT [9]. HSP90 exists in an activated form in tumor cells, while existing in a latent inactive form in normal cells [10,11]. Thus, HSP90 may serve as a unique target for cancer therapeutics.

HSP90 was shown to be expressed at higher levels in invasive breast cancer tissues compared with adjacent non-cancer tissues [12] and higher HSP90 expression correlates with worse prognosis [13]. Similarly, increased HSP90 protein expression was significantly associated with lymphatic invasion, lymph node metastasis, advanced stage, and poor survival in patients with gastric cancer [14]. HSP90 interacts with its client protein STAT3 and facilitates the binding of STAT3 to the *TWIST1* promoter, leading to the transactivation of TWIST1 and EMT induction in ovarian and renal cancer cells [15]. HSP90 triggers EMT in colorectal cancer cells through sustained activation of the NF-κB signaling pathway, and subsequent downregulation of the epithelial marker E-cadherin and upregulation of the mesenchymal marker Vimentin [16]. HSP90 physically associates with Oct-4 (a key regulator of stem cell pluripotency and differentiation) and prevents it from degradation by the ubiquitin-proteasome pathway in mouse embryonic stem cells [17].

Multiple gene expression data sets in the Cancer Genome Atlas (TCGA) indicated the upregulation of HSP90 in tissues of different cancer types (including cervical cancer) compared to the corresponding normal tissues [18,19]. High HSP90 expression is associated with poor prognosis in patients with head and neck cancer or colorectal cancer [18,19]. HSP90 protein was detected in cervical intraepithelial neoplasia [20]. The levels of HSP90 increased gradually from the normal cervix to intraepithelial lesions, and consequently to cervical cancer tissues [21]. Although the pharmacological inhibition of HSP90 inhibited the growth of cervical cancer cells [22], the exact function of HSP90 in cervical cancer development is still unclear.

Besides protein-coding RNAs, there are several known types of non-coding RNAs, including long non-coding RNAs (lncRNAs), circular RNAs, and microRNAs (miRNAs). Previous works have shown that all these non-coding RNAs are involved in the tumorigenesis and metastasis of human cancers [23]. MiRNAs function primarily as post-transcriptional regulators of mRNA by inhibiting the translation of their respective RNA targets or degrading their targets and show involvement in each of the cancer hallmarks [24,25]. LncRNAs can act as molecular sponges of miRNA, thereby affecting the expression of target genes of miRNAs [26]. The reduction in miR-361 expression in diverse tumor types and its tumor-suppressing function has been described [27]. However, previous studies exploring the role of miR-361 in cervical cancer produced somewhat controversial results: an early study suggested that miR-361 was downregulated in cervical cancer tissues and reduced miR-361 expression was sufficient to promote cervical cancer cell proliferation [28], whereas another study indicated that increased miR-361 expression was detected in patients with lymph node metastasis and stromal invasion and introduction of miR-361 facilitates cervical cancer progression [29]. Nevertheless, the precise role of miR-361 in cervical cancer and the mechanisms underlying its function in EMT have not fully explored.

Here, we showed that miR-361 expression was downregulated in cervical cancer tissues and cell lines, and directly targets HSP90 to inhibit the invasion and EMT features of cervical cancer cells. Furthermore, we discovered that NEAT1 functions as an oncogenic lncRNA that directly suppresses miR-361 expression and induces EMT and sphere formation in cervical cancer cells. Collectively, we have described a previously uncharacterized role for the NEAT1/miR-361/HSP90 signaling pathway in the regulation of cervical cancer development.

## 2. Materials and Methods 

### 2.1. Cell Lines 

Human cervical cancer cell lines (HeLa and SiHa) were obtained from ATCC. The normal endometrial epithelial cell line EM has been previously described [30]. The cells were cultured in DMEM/F12 medium (Gibco Laboratories, Grand Island, NY, USA) supplemented with 10% fetal bovine serum (Gibco Laboratories).

### 2.2. Cell Transfections 

The miR-361 mimic, control mimic, miR-361 inhibitor, control inhibitor, siRNA targeting HSP90 and NEAT1, as well as control siRNA, were purchased from Ambion (Austin, TX, USA). Expression vector encoding HSP90AA1 and the corresponding empty control vector were obtained from OriGene (Rockville, MD, USA). Cell transfections were performed using Lipofectamine 2000 (Invitrogen, Carlsbad, CA, USA) according to the manufacturer’s protocol.

### 2.3. Quantitative Real-Time PCR Assay 

Total RNA was extracted from cervical cancer cells and EM cells using TRIzol (Invitrogen). Total RNA was reverse transcribed into cDNA using a Reverse Transcription Kit (Takara, Shiga, Japan). Real-time PCR was performed using SYBR Green Real-Time PCR MasterMix (Toyobo, Osaka, Japan) using the ABI 7500 Real-Time PCR Systems, and GAPDH was used as the reference. The primers used in this study (except NEAT1) were obtained from the PrimerBank Web-based database (http://pga.mgh.harvard.edu/primerbank/). The primers to detect NEAT1 expression have been previously reported [31]. The NCode miRNA qRT–PCR kit (Invitrogen) was used to detect the miR-361 expression according to the manufacturer’s instructions. U6 small nuclear RNA was used as the internal normalization control. Data are expressed as the fold change over control (set as 1). The color-coded scale was used to represent the level of expression of genes in cervical cancer cells after overexpression or knockdown of miR-361 (or HSP90). 

### 2.4. Cell Proliferation Assay 

Cell proliferation was measured using the Cell Counting Kit-8 assay according to the manufacturer’s protocol (Dojindo Laboratories, Kumamoto, Japan). Briefly, cells (2 × 10^3^ each well) were seeded in a 96-well plate at 37 °C. Total of 10 μL CCK-8 solution was added to each well and plates were incubated at 37 °C for 2 h. Then, the absorbance was measured at 450 nm for each sample. The cell proliferation curves were plotted using the absorbance at each time point. 

### 2.5. Cell Invasion Assay 

Cell invasion assay was performed using 24-well transwell chambers (Corning Costar, Cambridge, MA, USA) as we previously described [3].

### 2.6. Cell Sphere Formation Assay 

Mammospheres were grown for 14 days as we previously reported [3]. Spheres larger than 50 mm were counted. 

### 2.7. Western Blot Assay 

Total cell lysates were extracted using the M-Per Mammalian Protein Extraction Reagent (Thermo Fisher Scientific, Waltham, MA, USA). The protein concentration was measured using a BCA protein assay kit (Thermo Fisher Scientific). Extracted proteins were separated by SDS-PAGE and transferred to a PVDF membrane. The membrane was blocked with 5% non-fat milk and incubated the primary antibodies for total HSP90 (AA1 and AB1, #4877, 1:1000), E-Cadherin (#5296, 1:1000), Vimentin (#5741, 1:1000) and GAPDH (#5174, 1:5000). These antibodies were purchased from Cell Signaling Technology (Danvers, MA, USA). GAPDH was used as an endogenous control. Immunoblot images were digitized and quantified using the ImageJ software (Wayne Rasband, NIH, USA). 

### 2.8. Luciferase Reporter Assay 

The 3′-untranslated region (3′-UTR) of HSP90 mRNA containing the putative miR-361 binding site was amplified using PCR and the resulting products were cloned downstream of the firefly luciferase gene in the pGL3 luciferase reporter vector (Promega, Madison, WI, USA). Mutations of the miR-361 binding site in the HSP90 3′-UTR (TCTGATA to CACAGCG) were generated by PCR mutagenesis using a QuickChange site-directed mutagenesis kit (Stratagene, La Jolla, CA, USA) according to the manufacturer’s instructions. The pGL3 luciferase reporter vectors containing wild-type (WT) NEAT1 fragment, or mutated (MUT) NEAT1 fragment with the mutated miR-361 binding site, were obtained from IGEbio (Guangzhou, China). Luciferase reporter assay was performed as we previously described [32]. In brief, cervical cancer cells were seeded into 24-well plates, and co-transfected after 24 h with the above luciferase reporter vectors (100 ng) containing HSP90 3′-UTR (WT or MUT) or NEAT1 (WT or MUT) and miR-361 mimic, miR-361 inhibitor or their respective controls (30 nM) using Lipofectamine 2000 (Invitrogen), along with the Renilla luciferase plasmid (10 ng, pRL-CMV, Promega) used for normalization. Two days after transfection, the luciferase activity was determined using a Dual-Luciferase Reporter assay (Promega) according to the manufacturer’s instructions. 

### 2.9. Statistical Analysis 

All results were expressed as mean ± SEMs of at least three independent experiments. Statistical analysis was conducted using 2-tailed Student’s t-test or 1-way ANOVA using the SPSS version 16.0 software (SPSS, Chicago, USA). *p*-values of < 0.05 were considered to be statistically significant. 

## 3. Results 

### 3.1. Downregulation of miR-361 Was found in Cervical Cancer 

**Figure 1:**
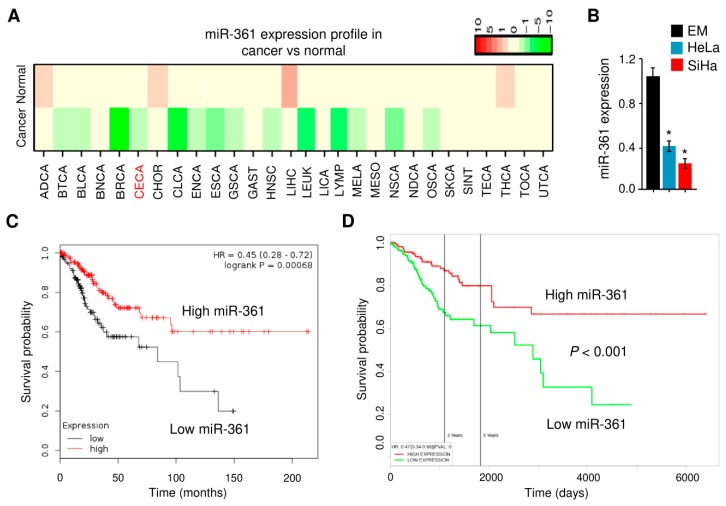
MiR-361 is aberrantly downregulated in cervical cancer tissues and cell lines. (**A**) Heatmap results showing differential expression patterns of miR-361 in different tumors and normal samples. MiR-361 expression in various types of cancer was searched in the dbDEMC v2.0 database. Green and red indicate downregulated and upregulated miR-361 expression in the corresponding cancer tissues, respectively. CECA: cervical cancer. (**B**) MiR-361 expression was measured in a human endometrial cell line (EM) and two human cervical cancer cell lines (HeLa and SiHa) using quantitative real-time PCR analysis. (**C**) The clinical significance of miR-361 expression in cervical cancer was evaluated by Kaplan–Meier survival analysis. The plots were generated using the KM-Plotter database. (**D**) The prognostic impact of miR-361 on cervical cancer was analyzed using the PROGmiR database. * *p* < 0.05.

We first evaluated the expression pattern of miR-361 across different normal and cancer tissues using the dbDEMC v2.0 database [33] and found that miR-361 was downregulated in almost all cancer types, including cervical cancer (Figure 1A). We then measured the miR-361 expression in one immortalized but non-malignant human endometrial cell line (EM) and in two human cervical cancer cell lines (HeLa and SiHa) using quantitative real-time PCR analysis. The expression of miR-361 in HeLa and SiHa cells was significantly lower than that in the EM cells (Figure 1B). Our Kaplan-Meier analysis using the KM-Plotter database [34] showed that decreased miR-361 expression level indicated a worse overall survival outcome in cervical cancer patients from the TCGA cohort (Figure 1C). The prognostic impact of miR-361 on cervical cancer patients was also analyzed using the PROGmiR database [35]. Survival analysis revealed lower expression of miR-361 was significantly associated with poorer prognosis in cervical cancer (Figure 1D). These results suggested that miR-361 was indeed downregulated in cervical cancer and its expression was positively associated with patient survival.

### 3.2. MiR-361 Inhibits EMT and Sphere Formation in Cervical Cancer Cells 

**Figure 2:**
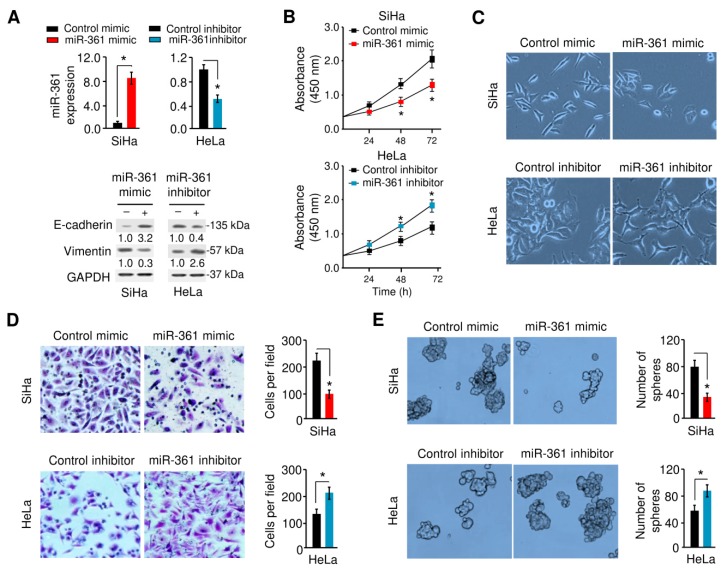
MiR-361 inhibits epithelial-mesenchymal transition (EMT) and sphere formation in cervical cancer cells. (**A**) Upper panel: MiR-361 expression was examined in SiHa cells transfected with miR-361 mimic or control mimic, and in HeLa cells transfected with miR-361 inhibitor or control inhibitor using quantitative real-time PCR analysis. Lower panel: Representative Western blot images for E-cadherin, Vimentin, and GAPDH protein levels in cervical cancer cells after overexpression or knockdown of miR-361. Numbers denote fold change of the E-cadherin/GAPDH or Vimentin/GAPDH protein ratio in miR-361 mimic-transfected SiHa cells versus control mimic-transfected cells (set as default 1), or in miR-361 inhibitor-transfected HeLa cells versus control inhibitor-transfected cells (set as default 1). (**B**) Growth curves of cervical cancer cells transfected as indicated were determined using CCK-8 assays. (**C**) MiR-361 affects cervical cancer cell morphology. (**D, E)** The effects of miR-361 expression on cell invasion (**D**) and sphere formation (**E**) were assessed using the Matrigel invasion and sphere formation assays. * *p* < 0.05.

We assessed the effects of miR-361 overexpression/knockdown on the proliferation, invasion, EMT, and sphere formation of SiHa/HeLa cells, which express relatively lower/higher levels of miR-361 (Figure 1B and Figure 2A). As shown in Figure 2B, the overexpression of miR-361 significantly attenuated the proliferation of SiHa cells, whereas the silencing of miR-361 markedly enhanced the growth of HeLa cells. Importantly, the overexpression of miR-361 induced the morphological changes in SiHa cells, from spindle-shaped appearance to cobblestone-like epithelial phenotype (Figure 2C). MiR-361-silenced HeLa cells become elongated and disassociated from their neighboring cells (Figure 2C). 

The marked effects of miR-361 expression on cell morphology led us to examine its influence on cell invasion and cancer stem cell-like properties using the Matrigel invasion assays and sphere formation assays. As shown in Figure 2D,E, the overexpression of miR-361 significantly decreased cell invasion and sphere formation, and miR-361 knockdown greatly induced these phenotypes. To investigate the mechanism by which miR-361 modulates EMT, we assessed the mRNA levels of known EMT markers, EMT-inducer genes and the drug resistance-associated gene utilizing quantitative real-time PCR analysis. The overexpression of miR-361 in SiHa cells increased the expression of two epithelial markers (*E-cadherin* and *ZO-1*) and reduced the expression of the mesenchymal marker Vimentin as well as *Twist1*, *MCL-1*, *Oct-4*, *SOX-2,* and *MDR-1* (Supplemental Figure S1A). In contrast, silencing of miR-361 led to opposite effects on these phenotypes in HeLa cells (Supplemental Figure S1A). Our Western blot analysis showed that enforced overexpression of miR-361 led to increased E-cadherin expression and decreased Vimentin expression (Figure 2A, lower panel). On the other hand, the inhibition of miR-361 reduced the expression of E-cadherin, and enhanced the levels of Vimentin (Figure 2A, lower panel). Collectively, these results suggested that miR-361 negatively regulates EMT and sphere formation in cervical cancer cells. 

### 3.3. Identification and Functional Annotation of Potential miR-361 Target Genes 

**Figure 3:**
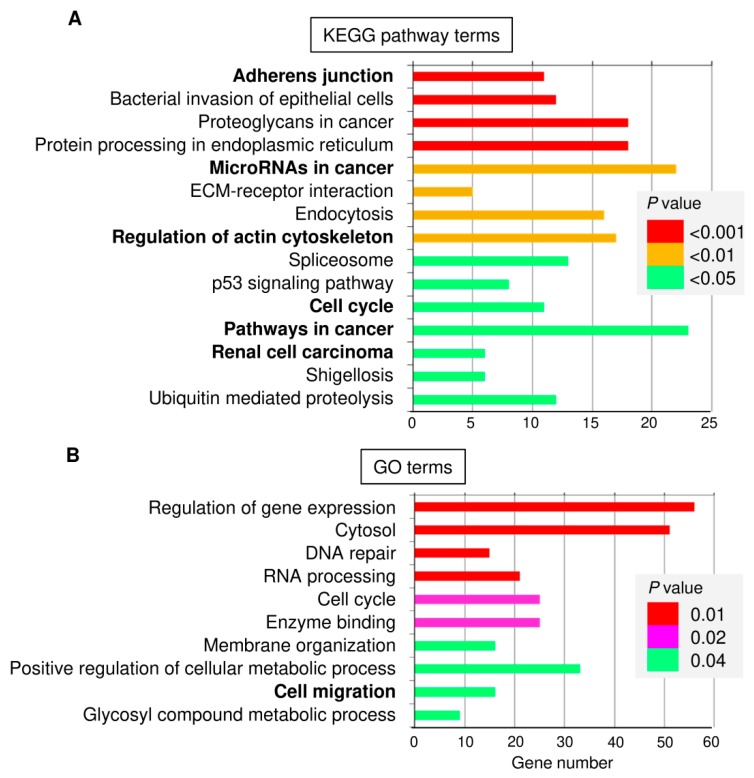
KEGG pathway and GO enrichment analysis for predicted miR-361 target genes using the DAVID database. (**A**) Enrichment analysis for KEGG pathways. (**B**) GO analysis according to the biological process.

To elucidate the relevant miR-361 target genes in cervical cancer, we predicted the potential target genes of miR-361 using three existing miRNA-target prediction programs, namely Targetscan [36], microRNA.org [37], and miRTarBase [38]. Targetscan and microRNA.org are the up-to-date sequence-based target prediction algorithms that consider the conservation of miRNAs [39]. miRTarBase is an up-to-date tool containing information on experimentally validated miRNA-target interactions [38]. Then we combined the prediction data resulting from these databases, and identified 160 overlapping genes as putative targets of miR-361. The web-based Database for Annotation, Visualization and Integrated Discovery (DAVID 6.7, https://david.ncifcrf.gov/) was used to provide the biological functional interpretation of the predicted targets of miR-361. KEGG pathway enrichment analysis revealed that these genes were significantly enriched in several pathways, including adhesion junction, microRNAs in cancer, regulation of actin cytoskeleton, cell cycle, pathways in cancer and renal cell carcinoma (Figure 3A). The top significantly biological process GO terms were involved in the regulation of gene expression, DNA repair, cell cycle, membrane organization, and cell migration (Figure 3B). 

**Figure 4:**
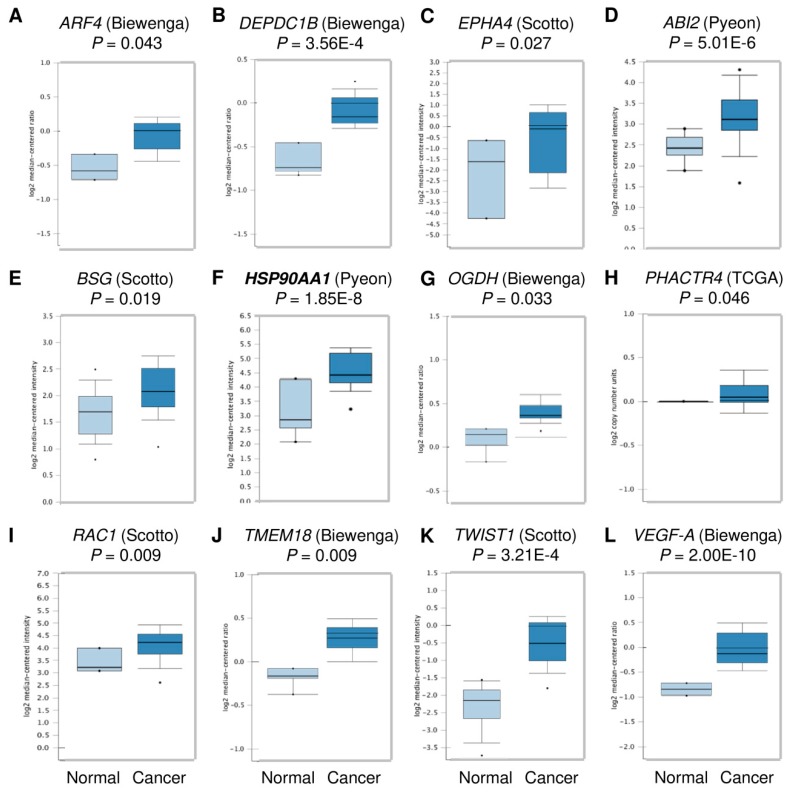
Box plots representing the expression level of the predicted miR-361 target genes in cervical.cancer tissues and normal cervical tissues (Oncomine database). (**A**) *ARF4* expression from Biewenga dataset, (**B**) *DEPDC1B* expression form Biewenga dataset, (**C***) EPHA4* expression from Scotto dataset, (**D**) *ABI2* expression from Pyeon dataset, (**E**) *BSG* expression from Scotto dataset, (**F**) *HSP90AA1* expression from Pyeon dataset, (**G**) *OGDH* expression from Biewenga dataset, (**H**) *PHACTR4* expression from the TCGA dataset, (**I**) *RAC1* expression from Scotto dataset, (**J**) *TMEM18* expression from Biewenga dataset, (**K**) *Twist1* expression from Scotto dataset, and (**L**) *VEGF-A* expression from Biewenga dataset.

**Figure 5:**
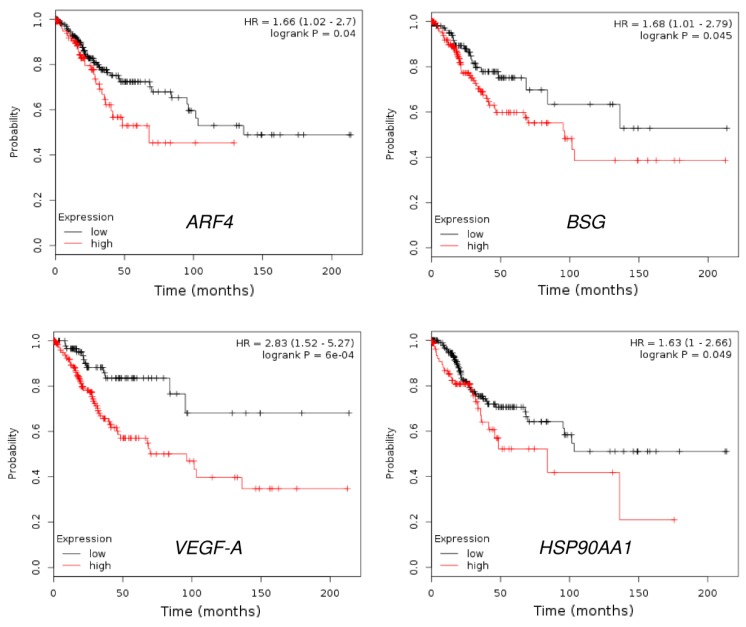
The prognostic value of *ARF4*, *BSG*, *VEGF-A,* and HSP90 expression in cervical cancer (KM-Plotter database).

Considering the critical role of miR-361 in regulating cervical cancer cell invasion, we decided to focus on the 16 predicted genes belonging to the “Cell migration” GO term category, including *HSP90AA1*, *ARF4*, *DEPDC1B*, *EPHA4*, *ABI2*, *BSG*, *GPC6*, *OGDH*, *PHACTR4*, *POMGNT2*, *RAC1*, *SEMA6D*, *TMEM18*, *VEGF-B*, as well as *Twist1* and *VEGF-A* (two known miR-361 targets) [40,41]. We explored their expression in human cervical cancer tissues and normal cervical tissues using the Oncomine database (https://www.oncomine.org/resource/login.html). Interestingly, among these 16 genes, 12 genes were significantly upregulated in cervical cancer tissues compared with normal tissues (Figure 4). We further performed the Kaplan-Meier analysis according to the mRNA expression of these genes using the KM-Plotter database. Consistent with a previous study showing that VEGF-A was an indicator of poor survival in patients with cervical cancer [42], high *VEGF-A* expression negatively correlated with overall survival (Figure 5). Our results also demonstrated that increased expression of *ARF4*, *BSG,* and HSP90 was significantly associated with shorter overall survival time in cervical cancer patients (Figure 5), suggesting that these genes might act as oncogenes in cervical cancer, and serve as potential biomarkers for predicting the prognosis of cervical cancer patients. 

### 3.4. HSP90 Is Overexpressed in Cervical Cancer Tissues 

**Figure 6:**
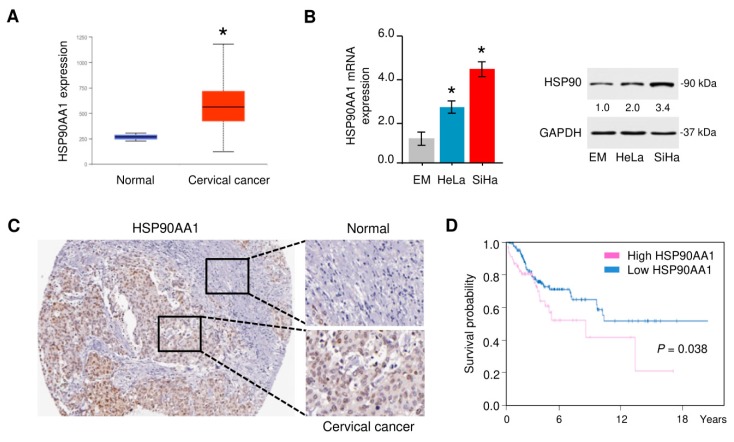
HSP90 is highly expressed in cervical cancer tissues. (**A**) The expression of HSP90 in cervical cancer tissues and normal tissues (UALCAN database). (**B**) Left panel: HSP90 expression was measured in a human endometrial cell line (EM) and two human cervical cancer cell lines (HeLa and SiHa) using quantitative real-time PCR analysis. Right panel: representative Western blot images for HSP90 and GAPDH protein levels in cervical cancer cell lines and normal EM cells. Numbers denote fold change of the HSP90/GAPDH protein ratio in cervical cancer cell lines versus normal EM cells (set as default 1). (**C**) HSP90 protein expression was upregulated in cervical cancer tissues compared with adjacent normal tissues (Human Protein Atlas database). (**D**) The prognostic value of HSP90 expression in cervical cancer (Human Protein Atlas database). * *p* < 0.05.

Since HSP90, one potential miR-361 target could modulate EMT of cancer cells [15,16], we selected this gene to determine whether miR-361 inhibits the EMT phenotypes through targeting HSP90. We first estimated the expression pattern of HSP90 in TCGA cervical cancer tissues and normal samples using the UALCAN web server [43]. We found that HSP90 expression was significantly higher in cervical cancer tissues than that in normal cervical tissues (Figure 6A). After that, the mRNA expression of HSP90 was measured in HeLa and SiHa cells compared with the normal endometrial cell line EM using quantitative real-time PCR and Western blot analysis, and HSP90 was significantly upregulated in cervical cancer cells (Figure 6B). To further verify the protein expression of HSP90 in cervical cancer tissues, we extracted the immunohistochemical staining images from the Human Protein Atlas database [44]. We verified that HSP90 expression in cervical cancer tissues was increased compared with adjacent normal cervical tissues (Figure 6C). Based on the data from the Human Protein Atlas database, cervical cancer patients with higher HSP90 expression had worse overall survival than those with lower HSP90 expression (Figure 6D). Overall, these findings support the relevance of HSP90 for cervical cancer carcinogenesis and progression. 

### 3.5. MiR-361 Binds to HSP90 and Suppresses Its Expression 

**Figure 7:**
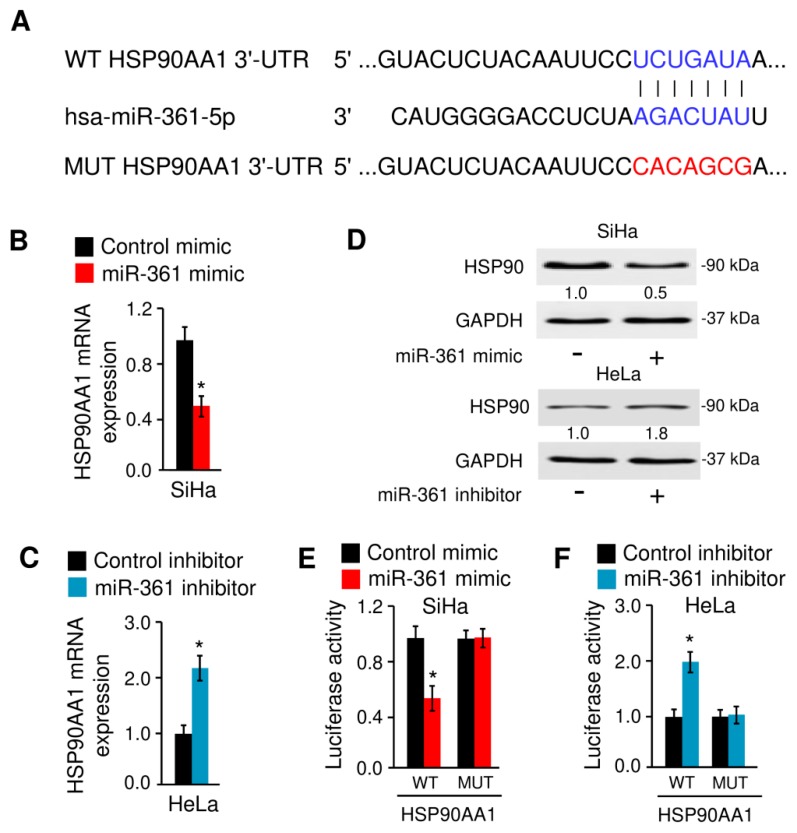
MiR-361 binds to HSP90 mRNA and suppresses its expression. (**A**) Illustration of the predicted binding site between miR-361 and HSP90 mRNA, with mutant sites shown in red. (**B**) The mRNA expression of HSP90 in SiHa cells transfected with miR-361 mimic or control mimic. (**C**) The mRNA level of HSP90 in HeLa cells transfected with miR-361 inhibitor and control inhibitor. (**D**) Western blot analysis of HSP90 and GAPDH expression in cervical cancer cells transfected as indicated. Numbers denote fold change of the HSP90/GAPDH protein ratio in miR-361 mimic-transfected SiHa cells versus control mimic-transfected cells (set as default 1), or in miR-361 inhibitor-transfected HeLa cells versus control inhibitor-transfected cells (set as default 1). (**E**) Luciferase assays in SiHa cells transfected with wild-type (WT) or mutant (MUT) HSP90 3′-UTR, together with miR-361 mimic or control mimic. (**F**) Luciferase assays in HeLa cells transfected with WT or MUT HSP90 3′-UTR, together with miR-361 inhibitor or control inhibitor. * *p* < 0.05.

Since the 3′-UTR of HSP90 (but not HSP90AB1) mRNA contains a sequence complementary to the seed regions of miR-361 (Figure 7A), we examined whether miR-361 regulates the HSP90 expression in cervical cancer cells. Ectopic expression of miR-361 could decrease, while inhibition of miR-361 could increase the mRNA and protein expression of HSP90 in cervical cancer cells (Figure 7B–D). To determine whether HSP90 is a direct target of miR-361, the pGL3 luciferase reporter vector containing the WT HSP90 3′-UTR or the MUT HSP90 3′-UTR were transfected into cervical cancer cells, together with miR-361 mimic, miR-361 inhibitor, or the respective negative controls, respectively. Luciferase reporter assays showed that overexpression of miR-361 could significantly decrease the luciferase activity of the WT HSP90 3′-UTR, but not the luciferase activity of the MUT HSP90 3′-UTR in SiHa cells (Figure 7E). Moreover, we found that the transfection with miR-361 inhibitor markedly induced the luciferase activity of the WT HSP90 3′-UTR in HeLa cells (Figure 7F). However, no obvious induction of luciferase activity was observed in HeLa cells transfected with miR-361 inhibitor and the luciferase reporter vector harboring the MUT HSP90 3′-UTR (Figure 7F). These results provided evidence that miR-361 directly reduces HSP90 expression in cervical cancer cells. 

### 3.6. HSP90 Enhances EMT and Sphere Formation Ability in Cervical Cancer Cells 

**Figure 8:**
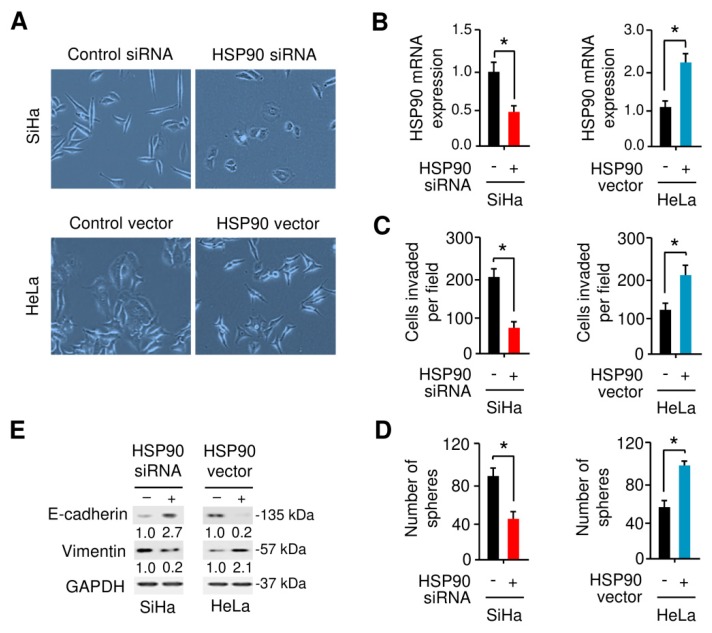
HSP90 enhances EMT and sphere formation in cervical cancer cells. (**A**) The morphology of.SiHa and HeLa cells transfected as indicated are shown. (**B**) Quantitative real-time PCR analysis of HSP90 mRNA expression was examined in SiHa and HeLa cells transfected as indicted. (**C**,**D**) The impact of HSP90 expression on cell invasion (**C**) and sphere formation (**D**) were assessed using the Matrigel invasion assay and sphere formation assays. (**E**) Representative Western blot images for E-cadherin, Vimentin, and GAPDH protein levels in cervical cancer cells after overexpression or knockdown of HSP90. Numbers denote fold change of the E-cadherin/GAPDH or Vimentin/GAPDH protein ratio in HSP90 siRNA-transfected SiHa cells versus control siRNA-transfected cells (set as default 1), or in HSP90 vector-transfected HeLa cells versus control vector-transfected cells (set as default 1). * *p* < 0.05.

We then examined whether HSP90 induces EMT properties in cervical cancer cells by observing cell morphology. As shown in Figure 8A,B, SiHa cells exhibited morphological changes from a spindle shape to a cobblestone-like shape upon knockdown of HSP90. On the other hand, the overexpression of HSP90 in HeLa cells altered the epithelial-looking morphology resulting in mesenchymal-like cells, and triggered cell scattering (Figure 8A,B). We also found that knockdown of HSP90 significantly inhibited the ability of SiHa cells to invade, and forced expression of HSP90 promoted the invasion of HeLa cells (Figure 8C). Since EMT and stem cell properties are interconnected, we further assessed the impact of HSP90 expression on cancer stem cell properties by performing sphere formation assays. As expected, HSP90-depleted SiHa cells partially lost the ability to form spheres compared with control cells, and the overexpression of HSP90 in HeLa cells induced sphere formation by approximately two-fold (Figure 8D). Our quantitative real-time PCR analysis confirmed that knockdown of HSP90 in SiHa cells significantly reduced the expression of mesenchymal marker *Vimentin* and the levels of *Twist1*, *MCL-1*, *Oct-4*, *SOX-2*, *and MDR-1*, but increased the expression of epithelial markers (*E-cadherin* and *ZO-1*) (Supplemental Figure S1B). However, HSP90 overexpression in HeLa cells has the opposite effects on these genes (Supplemental Figure S1B). As shown in Figure 8E, the silencing of HSP90 enhanced the protein expression of E-cadherin and reduced the protein expression of Vimentin, while overexpression of HSP90 decreased E-cadherin expression and increased Vimentin expression. Collectively, these findings indicated that HSP90 plays a critical role in promoting EMT and sphere formation in cervical cancer cells. 

### 3.7. MiR-361 Suppresses EMT and Sphere Formation in Cervical Cancer Cells by Inhibiting HSP90 Expression 

**Figure 9:**
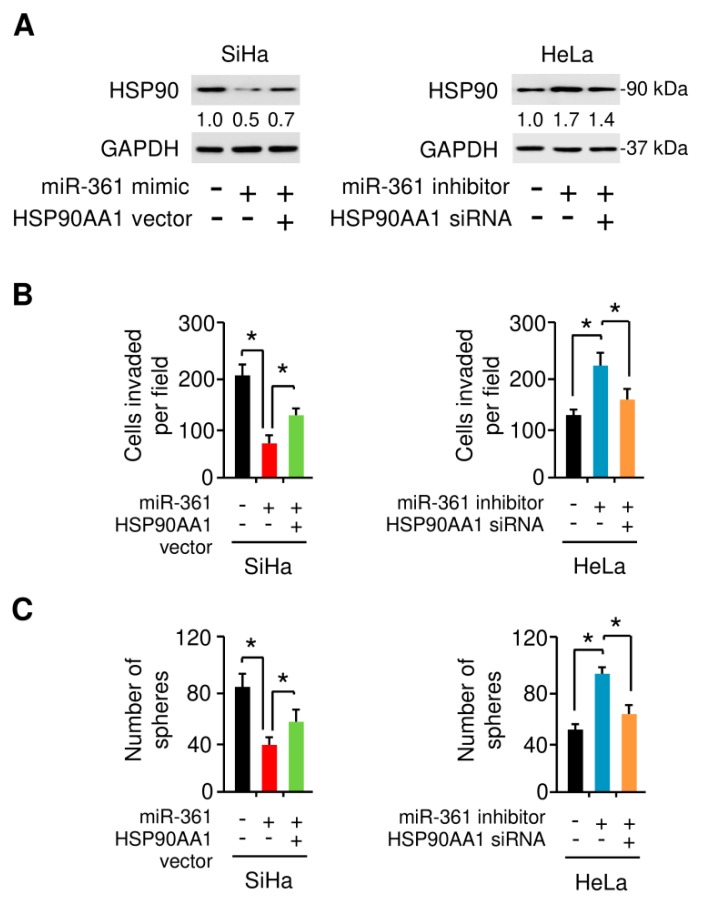
MiR-361 suppresses EMT and sphere formation in cervical cancer cells by inhibiting HSP90 expression. (**A**) HSP90 and GAPDH protein level in SiHa cells transfected with miR-361 mimic (or control mimic), together with (or without) HSP90 vector, and in HeLa cells transfected with miR-361 inhibitor (or control inhibitor), together with (or without) HSP90 siRNA. Numbers denote fold change of the HSP90/GAPDH protein ratio in cervical cancer cells transfected as indicated. (**B**,**C**) Cell invasion (**B**) and sphere formation (**C**) capacity was detected in cervical cancer cells transfected as indicated. * *p* < 0.05.

To examine the possibility that HSP90 is essential for miR-361-suppressed EMT and sphere formation in cervical cancer cells, we transfected SiHa cells with miR-361 mimic or control mimic, together with HSP90 expression vector or control vector (Figure 9A). The overexpression of HSP90 largely abolished the inhibitory effects of miR-361 on SiHa cell invasion and sphere formation (Figure 9B,C). To better characterize whether HSP90 expression is required for miR-361 inhibitor-induced HeLa cell invasion and sphere formation, we co-transfected HeLa cells with or without miR-361 inhibitor, together with HSP90 siRNA or control siRNA (Figure 9A). Indeed, we found that the promotion of cell invasion and sphere formation by knockdown of miR-361 could be suppressed by the inhibition of HSP90 expression (Figure 9B,C). These results suggested that decreased HSP90AA1 expression mediates the tumor-suppressive effects exerted by miR-361. 

### 3.8. NEAT1-Mediated miR-361 Downregulation Contributes to EMT and Sphere Formation of Cervical Cancer Cells Via Increasing HSP90 Expression 

**Figure 10:**
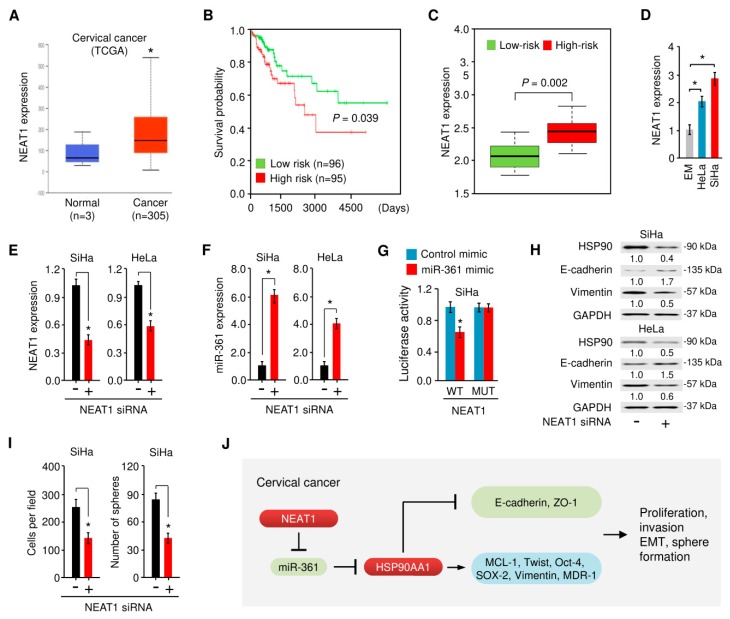
NEAT1-mediated miR-361 downregulation contributes to EMT and sphere formation of cervical cancer cells via increasing the HSP90 expression. (**A**) The expression of NEAT1 in cervical cancer tissues and normal tissues (UALCAN database). (**B**) The patients from the TCGA cervical cancer dataset in the SurvExpress database were divided into low- and high-risk groups and survival differences between the two groups were demonstrated with Kaplan-Meier survival curves. (**C**) The expression level of NEAT1 in low- and high-risk groups. (**D**) NEAT1 expression was examined in a normal endometrial cell line (EM) and in cervical cancer cell lines (HeLa and SiHa) using quantitative real-time PCR analysis. (**E**, **F**) NEAT1 (**E**) and miR-361 (**F**) expression in SiHa and HeLa cells transfected with NEAT1 siRNA or control siRNA. (**G**) Luciferase activities were measured in SiHa cells co-transfected with the reporter vectors (wild-type or mutant *NEAT1*), together with (or without) miR-361 mimic. (**H**) Western blot analysis of HSP90, E-cadherin, Vimentin, and GAPDH expression in SiHa and HeLa cells transfected with NEAT1 siRNA or control siRNA. Numbers denote fold change of the HSP90/GAPDH, E-cadherin/GAPDH or Vimentin/GAPDH protein ratio in NEAT1 siRNA-transfected cervical cancer cells versus control siRNA-transfected cells (set as default 1). (**I**) Cell invasion and sphere formation capacity were detected in SiHa cells transfected with NEAT1 siRNA or control siRNA. (**J**) A scheme illustrating the mechanism by which lncRNA NEAT1 sponges miR-361 to upregulate the expression of HSP90, thereby promoting EMT and cancer stem cell properties in cervical cancer cells. * *p* < 0.05.

Recent studies suggested that lncRNAs can regulate gene expression by competitive binding to miRNAs during cancer progression [26]. We have reported that the lncRNA NEAT1 functions as an oncogenic sponge for miR-361 in endometrial cancer [32], raising the possibility that NEAT1 potentially interacts with miR-361 and suppresses its expression in cervical cancer cells. To test this, we searched for NEAT1 expression in TCGA datasets from cervical cancer tissues and normal cervical tissues using the UALCAN database. The results revealed a significantly higher level of NEAT1 in cervical cancer samples compared with normal samples (Figure 10A).

We then assessed the association between NEAT1 expression and overall survival of patients with cervical cancer using the online databases SurvExpress [45], where cervical cancer patients from the TCGA dataset (*n* = 191) were divided into low- and high-risk groups for poor prognosis. As expected, the overall survival of patients with high-risk (red line) was significantly shorter compared with those with low-risk (green line) (Figure 10B). As shown in Figure 10C, the expression of NEAT1 in the high-risk group was significantly higher than that in the low-risk group. Additionally, the expression of NEAT1 was measured in cervical cancer cell lines compared with the normal cell line EM, and NEAT1 was significantly upregulated in cervical cancer cell lines (Figure 10D). 

We examined whether NEAT1 depletion influences miR-361 expression in cervical cancer cells using quantitative real-time PCR analysis. As shown in Figure 10E,F, the depletion of NEAT1 by siRNA caused the upregulation of miR-361 in cervical cancer cells. To verify the direct binding relationship between NEAT1 and miR-361, we performed a luciferase reporter assay by co-transfecting cervical cancer cells with luciferase reporter plasmids containing the WT *NEAT1* or the MUT *NEAT1*, along with miR-361 mimic or control mimic. Our results showed that the overexpression of miR-361 significantly decreased the luciferase activity driven by the WT *NEAT1*, but caused no significant change in the luciferase activity of the MUT *NEAT1* (Figure 10G). These results suggested that NEAT1 directly interacts with miR-361 and represses its expression in cervical cancer cells. 

We further examined the effects of NEAT1 depletion on the protein expression of HSP90 and found that the silencing of NEAT1 greatly suppressed the expression of HSP90 in cervical cancer cells (Figure 10H). Furthermore, Western blot analysis showed knockdown of NEAT1 could markedly increase the level of E-cadherin, but decrease the expression of Vimentin (Figure 10H). 

Through cell invasion and sphere formation experiments, we found that NEAT1 knockdown led to decreased cell invasion and sphere formation (Figure 10I). Taken together, these findings support the notion that NEAT1 promotes cervical cancer invasion and sphere formation through upregulating HSP90 expression via binding with miR-361, a tumor suppressor that directly suppresses HSP90 expression. 

## 4. Discussion 

Previous studies illustrating the important functions of miR-361 in the suppression of various malignant properties have established its widespread role in tumorigenesis and metastasis [27]. However, its ability to mediate cervical cancer initiation and progression is still controversial [28,29]. Consistent with an early report that revealed that overexpression of miR-361 in SiHa and HeLa cells has anti-proliferative effects by repressing the expression of its target gene FOXM1 [28], here our results suggested a role for miR-361 as a key suppressor in cell proliferation. Beyond that, our data showed that miR-361 serves as a novel regulator of EMT properties, thereby contributing to its tumor suppressor function. 

It has been previously shown that miR-361 appears to be a molecular hub to participate in the control of multiple cancer cell signaling networks [27]. Several reports have suggested that miR-361 plays a critical role in repressing tumor progression by targeting multiple components of many essential signaling pathways implicated in tumor growth, EMT, metastasis, drug resistance, glycolysis, angiogenesis, and inflammation, such as the PTEN/PI3K/AKT, Wnt/β-catenin, EMT/cancer stem cell-related pathways, and VEGF pathway [27]. These data, together with our findings, point to the complexity of miR-361-regulated signaling pathways that determine the phenotypes of human tumor cells. The detailed role of miR-361 in cervical cancer and molecular mechanisms underlying its action remain to be further clarified. 

HSPs, especially HSP90, are well-known for their critical roles in modulating cell proliferation, apoptosis, migration, invasion, EMT, cancer stem cell features, metastasis, and angiogenesis [7]. HSP90 could function as an EMT-inducer in ovarian, renal, and colon cancer [15,16]. Our computational screening and experimental validation have identified HSP90 as a key downstream target of miR-361 in cervical cancer cells. Our study provided new evidence that HSP90 promotes EMT through increasing the expression of Twist1 and MCL-1, which were demonstrated to accelerate the process of EMT and invasion of cervical cancer cells [46,47]. In addition, the expression of two cancer stem cell-related genes (*Oct-4* and *SOX-2*) was elevated in cervical cancer tissues compared with normal tissues [48,49], and their expression was significantly upregulated in HSP90-overexpressing cervical cancer cells (Supplemental Figure S1B), suggesting that loss of miR-361 expression elevates HSP90 levels, and causally increases the expression of Twist1, MCL-1, Oct-4, and SOX-2, leading to the acquisition of EMT phenotypes of cervical cancer cells. 

In addition to its intracellular localization, HSP90 is also a secreted and cell surface protein. Extracellular HSP90 promotes EMT and cancer cell invasion and stimulates metastatic spread [50], and blocking the secreted HSP90 significantly inhibits melanoma metastasis [51], indicating the possibility that HSP90 secretion might be involved in cervical cancer progression and metastasis. Therefore, the effects of miR-361 and NEAT1 expression on the secretion of HSP90 by cervical cancer cells should be explored using the enzyme-linked immunosorbent assay. Whether the secreted HSP90 acts as a pivotal regulator of cervical cancer metastasis requires further investigation. 

ARF4 (a member of the RAS superfamily) is a small guanine nucleotide-binding protein and overexpression of ARF4 has been observed in human tumors, including ovarian cancer, lung cancer, and glioma [52]. ARF4 serves as a positive regulator in breast cancer cell migration [53]. *BSG* (also known as *CD147* and *EMMPRIN*) is a glycosylated transmembrane protein that is overexpressed in cervical cancer [54]. Furthermore, increased BSG expression correlated significantly with poor survival in patients with cervical cancer [55]. BSG was thought to enhance tumor metastasis, angiogenesis, and drug resistance through its association with various proteins [56]. Our findings were consistent with these previous results, and showed that increased mRNA levels of *ARF4* and *BSG* were abnormally overexpressed in cervical cancer samples and its expression correlated with poorer patient prognosis. More importantly, our study suggested a possibility that miR-361 inhibits cervical cancer growth and metastasis by simultaneously targeting *ARF4* and *BSG*. Further investigations were needed to explore the functional link between miR-361 and ARF4/BSG in cervical cancer cells. 

The dysregulation of lncRNA NEAT1 exerts its oncogenic functions in the majority of human cancers [57]. NEAT1 was reported to be upregulated in cervical cancer tissues compared to adjacent normal tissues, and enhanced NEAT1 expression was significantly correlated with larger tumor size, poor differentiation, progressed Federation of Gynecology and Obstetrics stage, lymph node metastasis, and decreased survival rate, thus it represents as a prognostic marker for cervical cancer [58]. Similarly, the expression of NEAT1 was increased in cervical cancer samples compared with normal samples, and the patients expressing high NEAT1 levels showed a shorter survival time [59]. Recent mechanism experiments revealed that knockdown of NEAT1 inhibited cervical cancer development through repressing cell proliferation, migration, and invasion and also inducing cell apoptosis by regulating the miR-133a/SOX4 pathway [60]. Also, NEAT1 functioned as a molecular sponge for miR-9-5p to promote the proliferation and migration of cervical cancer cells [61]. It has been also clarified that knocking down the expression of NEAT1 in cervical cancer cells resulted in the repression of cell proliferation and invasion via the PI3K/AKT signaling pathway [62]. However, the study on the impact of NEAT1 on the EMT program and the self-renewal capacity of cervical cancer cells is limited. Here, we demonstrated that, by competitively binding to miR-361 and suppressing its expression, NEAT1 indirectly upregulates the expression of the EMT inducer HSP90, thereby promoting EMT, invasion and sphere formation of cervical cancer cells. Future investigation will be devoted to determining the mechanism by which NEAT1 performs this function in cervical cancer (especially the cross-talk between NEAT1 and other tumor suppressor miRNAs or the components of polycomb repressive complex 2). 

## 5. Conclusions 

We demonstrated that NEAT1 functions as an endogenous inhibitor of miR-361, thereby leading to the de-repression of miR-361 target HSP90 as well as EMT induction (Figure 10J). Our study described a previously unappreciated connection between the NEAT1/miR-361/HSP90 axis and EMT phenotypes in cervical cancer cells. These findings provided useful insights into the mechanisms of cervical cancer tumorigenesis and metastasis. 

## Supplementary Materials

The following are available online at https://www.mdpi.com/2073-4409/9/3/632/s1, Supplemental Figure S1: MiR-361 inhibits EMT in cervical cancer cells, Bioinformatics analysis: UALCAN database, Human Protein Atlas database.

## Abbreviations

CC	Cervical cancer
lncRNA	Long non-coding RNA
TCGA	The Cancer Genome Atlas
EMT	Epithelial-mesenchymal transition
