# Supplementary material for: MicroRNA-361-Mediated Inhibition of HSP90 Expression and EMT in Cervical Cancer Is Counteracted by Oncogenic lncRNA NEAT1
Source: Cells. 2020 Mar 5;9(3):632. doi: 10.3390/cells9030632 (PMC7140536; doi:10.3390/cells9030632)
Supplement: Supplementary file 1 [file cells-09-00632-s001.pdf]

Supplementary data

MicroRNA-361- Mediated Inhibition of HSP90 Expression and EMT in Cervical Cancer is Counteracted by Oncogenic lncRNA NEAT1

Daozhi Xu [1,†], Peixin Dong [1,†,*], Ying Xiong [2,†], Junming Yue [3,4], Yosuke Konno [1], Kei Ihira [1], Noriko Kobayashi [1], Yukiharu Todo [5] and Hidemichi Watari [1,*]

1. Department of Obstetrics and Gynecology, Hokkaido University School of Medicine, Hokkaido University, Sapporo, Japan; xudaozhi87@yahoo.co.jp (D.H.); dpx1cn@gmail.com (P.D.); konsuke013@gmail.com (Y.K.); ihey0610@huhp.hokudai.ac.jp (K.I.); norikingyo@med.hokudai.ac.jp (N.K.); watarih@med.hokudai.ac.jp (H.W.)
2. Department of Gynecology, State Key Laboratory of Oncology in South China, Sun Yat-sen University Cancer Center, Guangzhou, P. R. China; tdken999@163.com
3. Department of Pathology and Laboratory Medicine, University of Tennessee Health Science Center, Memphis, TN 38163, USA; jyue@uthsc.edu
4. Center for Cancer Research, University of Tennessee Health Science Center, Memphis, TN 38163, USA
5. Division of Gynecologic Oncology, National Hospital Organization, Hokkaido Cancer Center, Sapporo, Japan; yukiharu@sap-cc.go.jp

* Correspondence: dpx1cn@gmail.com (D.P); watarih@med.hokudai.ac.jp (H.W.)

Bioinformatics analysis

UALCAN database

The expression of *HSP90* in cervical cancer tissues and normal cervical tissues, and clinical information was extracted from the UALCAN database [43], which downloaded the level-3 TCGA RNA-sequencing data representing 31 cancer types from the TCGA website. The expression of *HSP90* was analyzed in cervical cancer tissues (n = 305) and normal cervical tissues (n = 3). The "raw_count" column shows the number of unfiltered fragments that are aligned with gene, and the "scaled_estimate" column provides the estimation of transcripts generated from *HSP90* gene. The "scaled_estimate" was multiplied by 10^6 to obtain transcripts per million (TPM) expression value using in-house PERL (Practical Extraction and Report Language) program. TPM expression value was used to estimate *HSP90* gene expression. Patient clinical parameters (such as age, sex, race, survival status, tumor grade, and tumor stage) were retrieved as XML (eXtensible Markup Language) files from Genomic Data commons (https://gdc.cancer.gov/). Integration processing of datasets was implemented using Perl (http://www.perl.org/) scripting tools. TCGA RNA-sequencing data corresponding to the primary cervical cancer and normal cervical samples for *HSP90* gene expression was represented as boxplots.

Human Protein Atlas database

We examined the HSP90 protein expression in human cervical cancer tissues and adjacent normal tissues using the Human Protein Atlas database [44], which provides data on the protein expression patterns of various cell types in both cancerous and normal tissues. HSP90 protein expression was evaluated using immunohistochemistry on cervical cancer samples tissue microarray (n = 24). This database also contains the TCGA RNA-sequencing data from different forms of human cancers, and the FPKMs (number Fragments Per Kilobase of exon per Million reads) for *HSP90* gene were subsequently used for quantification of *HSP90* expression in cervical cancer tissues. According

to the FPKM value of *HSP90* gene, cervical cancer patients were classified into two expression groups: patients with lower expression of *HSP90* (*n* = 208) and those with higher expression of *HSP90* (*n* = 83). The correlation between *HSP90* expression level and patient survival was examined using Kaplan-Meier survival estimators, and the survival outcomes of the two groups were compared by log-rank tests.

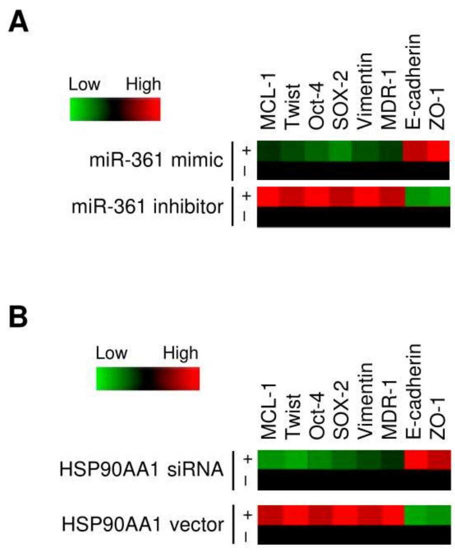

Supplemental Figure 1: MiR-361 inhibits EMT in cervical cancer cells. (**A**) The color-coded scale depicting expression changes of indicated mRNAs in cervical cancer cells after overexpression or knockdown of miR-361, as determined by quantitative real-time PCR analysis. Red: upregulation; green: downregulation. (**B**) The color-coded scale depicting expression changes of indicated mRNAs in cervical cancer cells after overexpression or knockdown of HSP90, as determined by quantitative real-time PCR analysis. Red: upregulation; green: downregulation.
